# Effect of Bariatric Surgery on Risk of Complications After Total Knee Arthroplasty: A Randomized Clinical Trial

**DOI:** 10.1001/jamanetworkopen.2022.6722

**Published:** 2022-04-14

**Authors:** Michelle M. Dowsey, Wendy A. Brown, Angela Cochrane, Paul R. Burton, Danny Liew, Peter F. Choong

**Affiliations:** 1 Department of Surgery, St Vincent’s Hospital Melbourne, The University of Melbourne, Fitzroy, Melbourne, Victoria, Australia; 2 Department of Orthopaedics, St Vincent’s Hospital Melbourne, Fitzroy, Melbourne, Victoria, Australia; 3 Department of Surgery, Central Clinical School, Monash University, Melbourne, Australia; 4 School of Public Health and Preventive Medicine, Monash University, Melbourne, Australia

## Abstract

**Importance:**

People with severe obesity who undergo a total knee arthroplasty (TKA) for osteoarthritis (OA) are at higher risk of short-term and long-term complications compared with people with reference (<30) body mass index (BMI; weight in kilograms divided by height in meters squared). It is not known whether weight loss before TKA modifies this risk.

**Objective:**

To determine whether outcomes are improved by undergoing bariatric surgery before TKA in people with BMI greater than or equal to 35 and end-stage OA.

**Design, Setting, and Participants:**

This parallel-group, assessor-blinded, randomized clinical trial was conducted between May 2012 and June 2020 with a minimum follow-up of 12 months after TKA. TKA was performed at a tertiary referral university-affiliated public hospital, and bariatric surgery was performed at a private hospital facility and a university-affiliated private practice. Data analysis was performed from February to July 2021.

**Interventions:**

Bariatric surgery compared with usual weight management advice (treatment as usual [TAU]) in people scheduled for TKA.

**Main Outcomes and Measures:**

The primary outcome was complications of TKA measured by a composite of death from any cause, perioperative or postoperative complications resulting in a discharge delay, unplanned procedure, or readmission for at least 12 months after TKA. Secondary outcomes included hospital bed day utilization, anthropomorphic measures, and patient-reported outcomes.

**Results:**

Eighty-two patients waiting for TKA were randomized to undergo bariatric surgery (41 patients) or TAU (41 patients). Of the 82 participants, 66 (80.5%) were women, the mean (SD) age was 57.8 (4.9) years, and the mean (SD) BMI was 43.8 (5.5). Thirty-nine participants (95.1%) in the intervention group underwent laparoscopic adjustable gastric banding, and 29 (70.7%) subsequently underwent TKA. Thirty-nine patients (95.1%) in the TAU group underwent TKA. Six patients (14.6%) in the intervention group incurred the primary outcome (median follow-up, 24 months), compared with 15 (36.6%) in the TAU group (median follow-up, 27 months) (difference, 22.0%; 95% CI, 3.7% to 40.3%; *P* = .02). The between-group difference in BMI at 12 months was −6.32 (95% CI, −7.90 to −4.50; *P* < .001) in favor of the intervention group. TKA was declined by 12 participants (29.3%) in the intervention group because of symptom improvement, whereas 2 participants (4.9%) in the TAU group declined TKA (difference, 24.4%; 95% CI, 9.0% to 39.8%; *P* = .003).

**Conclusions and Relevance:**

Weight loss following bariatric surgery reduced the risk of complications of TKA in people with BMI greater than or equal to 35. Significantly fewer participants required TKA following weight loss, contributing to this finding.

**Trial Registration:**

Australian New Zealand Clinical Trials Registry Number: ACTRN12611001178932

## Key Points

**Question:**

Does bariatric surgery reduce complication risk after total knee arthroplasty (TKA) in patients with severe obesity?

**Findings:**

In this randomized clinical trial of 82 adults aged 65 years and younger with class II obesity, those who were scheduled to undergo TKA experienced fewer joint surgery complications after undergoing a bariatric procedure, compared with those who underwent TKA without weight loss intervention.

**Meaning:**

The findings suggest that people with severe obesity and knee osteoarthritis should seek to lose weight before considering TKA, and weight loss appears to reduce the complications of joint surgery.

## Teaser

This randomized clinical trial examines whether outcomes are improved by undergoing bariatric surgery before total knee arthroplasty among individuals with severe obesity and end-stage osteoarthritis.

## Introduction

Worldwide, more than 240 million people have symptomatic and activity-limiting osteoarthritis (OA) of the hip or knee.^1^ More than 50% of people with knee OA will undergo a total knee arthroplasty (TKA) during their lifetime.^2^ Obesity is also among the most prevalent diseases,^3^ is a major factor associated with the genesis of OA,^4^ is associated with ongoing joint symptoms,^5^ and is overrepresented in people presenting for TKA.^6^

Short-term and long-term outcomes after TKA are known to be inferior in patients with severe obesity compared with patients in the reference weight range,^7,8,9,10,11,12,13^ yet it remains unclear whether there are potential benefits for patients if obesity is treated before TKA.^14^ Studies of diet-induced weight loss before TKA report improved outcomes compared with treatment as usual (TAU), but tend to include patients of lower body mass index (BMI; weight in kilograms divided by height in meters squared), achieve weight loss to a lesser degree than surgery-induced weight loss,^15^ and involve short-term follow-up.^16^

To our knowledge, no trial evidence exists of the efficacy of weight loss before TKA^17^ in people with severe obesity and advanced OA. This is despite an increase in the prevalence and degree of obesity observed in TKA recipients over time^18^ and evidence of higher in-hospital morbidity and mortality, length of stay, and direct hospital costs in patients with severe obesity.^19^

To address this important clinical question, we designed a randomized clinical trial (RCT) with the aim of determining whether the incidence of postoperative complications could be improved for patients with severe obesity and end-stage OA if TKA was preceded by substantial weight loss. We chose bariatric surgery as the weight loss intervention because it can reliably induce and sustain 15% to 30% total body weight loss beyond 10 years.^15,20^ This is more substantial and durable than any nonsurgical weight loss programs.^21^

We hypothesized that clinical, functional, and quality of life (QoL) outcomes in patients with severe obesity (BMI ≥35) undergoing TKA would be improved if it were preceded by a bariatric procedure. At the time our study commenced, laparoscopic adjustable gastric banding (LAGB) was the most performed procedure in our jurisdiction and was the bariatric procedure chosen for this study.

## Methods

### Study Centers and Trial Design

The study was conducted in Victoria, Australia, at St Vincent’s Hospital Melbourne (SVHM), a tertiary referral university-affiliated institution; The Avenue Hospital, a private 152-bed facility; and The Centre for Bariatric Surgery, a private bariatric practice affiliated with Monash University. A parallel-group, assessor-blinded RCT was conducted between May 2012 and June 2020, with a planned minimum follow-up of 12 months. The study was constructed and presented in accordance with Consolidated Standards of Reporting Trials (CONSORT) reporting guidelines for RCTs.^22^ The trial protocol (Supplement 1) was approved by the Human Research Ethics Committees of SVHM and The Avenue Hospital.

### Setting and Recruitment

Participants were recruited from an orthopedic clinic at SVHM between May 2012 and December 2016 after providing written informed consent. Inclusion criteria were age 65 years or younger, BMI greater than or equal to 35, being on the surgical waiting list for primary TKA, and being willing to cooperate in a long-term weight management program. Exclusion criteria were revision surgery, surgery for neoplastic disease, a medical condition that in the opinion of the investigators made the patient unsuitable for participation in the trial, previous esophagogastric surgery (eg, fundoplication), and lack of acceptance of the randomization process.

### Intervention

Patients randomized to the intervention were clinically assessed and provided consent for their bariatric procedure by 1 of 2 surgeons affiliated with the Centre for Bariatric Surgery (W.A.B. and P.R.B.). Surgery was preceded by 2 weeks of a weight-loss program (Optifast) to reduce liver size before placement of the LAGB using the Allergan Health Lap-Band System. LAGB was performed at The Avenue Hospital as either a day procedure or as an overnight stay if medically indicated. Patients underwent a routine barium-enhanced esophagogram before discharge to assess for position of the band. Patients attended regular follow-up visits after LAGB, as described elsewhere,^23^ in which lifelong follow-up is intended. In brief, 3 to 5 visits are scheduled at 2-week to 4-week intervals, reducing to 3-week intervals, then 6-month intervals, and, ultimately, patients are seen once per year, at a minimum. Clinical visits involve LAGB adjustment to optimize satiety without inducing adverse symptoms. Education and advice regarding eating behavior, dietary intake, and lifestyle change are provided at each visit. Patients returned to SVHM for TKA at 12 months or after a 20% loss of baseline body weight if this occurred earlier. In line with standard practice, patients in the comparator TAU group underwent TKA with routine follow-up and were provided general weight management advice. A standardized clinical pathway protocol was used for TKA and all follow-up care.^24^

### Outcomes

The primary outcome measure was a composite of the following at any time from TKA until the study close: death from any cause, perioperative or postoperative complications that result in a delay in discharge (eg, fracture, neurapraxia, sepsis, nosocomial infection, myocardial infarction, bowel obstruction, venous thromboembolism, cerebrovascular event, and renal failure), wound complications (eg, infections, hematomas, and dehiscence), prosthetic infection, and unplanned procedures and/or readmission. The process for identifying the primary outcome was conducted by a research officer (not a coauthor of this article) who was blinded to study group. Active surveillance for complications comprised review of all medical records on patient discharge and at each outpatient’s review, telephone calls to participants once every 3 months (conducted by A.C.), and review of records from other health care institutions as required. Information on possible primary outcomes of interest was reviewed by a blinded verification panel, which consisted of 2 orthopedic surgeons (not coauthors of this article) and a senior nurse specialist (M.M.D.).

Secondary outcomes were hospital bed day utilization; change in BMI and weight at 12 months after TKA; and pain, function, and QoL using the Western Ontario McMaster Universities Osteoarthritis Index^25^ and the Veterans Rand 12 item^26^ questionnaires. The questionnaires were administered at enrollment and at 12 months after TKA.

Data pertaining to LAGB were collected prospectively on a real-time web-based program (Lap-Base) at the Centre for Bariatric Surgery. At SVHM, all clinical data and patient questionnaires were captured in the hospital’s arthroplasty registry (SMART).^27^

### Sample Size

The sample size calculation was based on 2-sided α = .05, at 80% power, and expected rates of the primary outcome at 1 year after TKA of 8.8% for the intervention group and 29.6% for the TAU group. The expected rates were derived from existing data from the SMART Registry.^27^ A consecutive cohort of 529 patients who underwent TKA^28^ was extracted from the registry, of whom 118 patients were aged 65 years or younger with BMI of 35 or higher; 35 of 118 patients (29.6%) experienced the primary outcome. Hence, this was the assumed rate for the TAU group. LAGB was expected to reduce weight by 20% by 12 months. This amount of weight loss would have led to reclassification of most of the 118 patients to a BMI category of less than 35. Hence, the estimated rate of the primary outcome for the intervention group was derived from same cohort of 529 patients, of whom 137 had a BMI of less than 35; 12 of 137 patients (8.8%) experienced the primary outcome. The sample size required in each of the 2 groups was 55. To allow for dropout of patients, we aimed to recruit 120 patients in total. These numbers granted more than 80% power to detect the expected differences in cumulative event-free survival between the 2 groups. There were no planned interim analyses.

### Randomization and Blinding

A research coordinator (A.C.) was responsible for participant recruitment and consent. Participants were randomly assigned 1:1 using computer-generated random permuted blocks of 4 to 12 prepared in advance by an independent biostatistician (not a coauthor of this article) and stored in a password-protected file. Patient assignment was performed by a researcher (not a coauthor of this article) who had no direct contact with patients. Blinding of surgeons performing TKA was not feasible, but these surgeons had no role in outcome assessment.

### Statistical Analysis

All analyses were performed on an intention-to-treat basis in a blinded manner using Stata statistical software version 15.0 (StataCorp). The χ^2^ test (2-sided) was applied to assess the primary outcome, with significance set at *P* < .05. Survival analyses were undertaken using Cox proportional hazards regression to derive hazard ratios associated with intervention group compared with the TAU group. Mann-Whitney *U* tests were used to assess the secondary outcomes of hospital bed day utilization, and for anthropomorphic measures, pain, function and QoL. Data analysis was performed from February to July 2021.

## Results

### Participants

**Figure 1.**
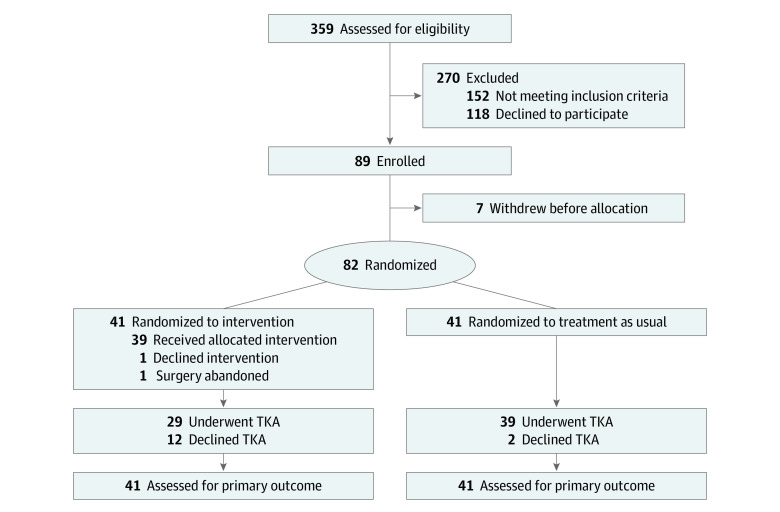
Flowchart of Screening, Randomization, and Follow-up of Study Participants TKA indicates total knee arthroplasty.

In total, 359 individuals were assessed for study eligibility between May 2012 and December 2016, of whom 277 were excluded and 118 declined to participate (Figure 1) for reasons outlined in the eTable in Supplement 2. Of 89 enrolled individuals, 7 subsequently withdrew before treatment allocation and 82 participants were randomized. Trial enrollment was terminated before reaching the planned sample size because it was apparent that the study would not be completed within an acceptable period. This was because a substantial portion of patients in the intervention group who also underwent bariatric surgery (12 of 39 patients [30.8%]) declined planned TKA because of symptom improvement. Overall, TKA was declined by 12 of 41 participants (29.3%) in the intervention group, whereas only 2 of 41 participants (4.9%) in the TAU group declined TKA (difference, 24.4%; 95% CI, 9.0% to 39.8%; *P* = .003). Given the delays to TKA, substantial alteration to the trial protocol would have been required to accrue the intended number of participants undergoing TKA. This decision was made without any formal assessment of outcomes; however, given the low number of individuals proceeding to TKA within 1 year of LAGB, we assumed the study’s power would still be sufficient to address the primary outcome. The final LAGB procedure was undertaken in March 2017 and final TKA in March 2019. The study was formally closed June 2020, at which time 12 participants who had received LAGB had continued to decline TKA because of symptom resolution.

Of 82 participants (41 in the intervention group and 41 in the TAU group), 66 (80.5%) were female, the mean (SD) age was 57.8 (4.9) years, and the mean (SD) BMI was 43.8 (5.5). Baseline characteristics were similar between groups (Table 1). Of the 41 patients allocated to the intervention, 39 (95.1%) underwent LAGB. One participant declined to undergo LAGB, and 1 case was abandoned because of hepatomegaly. Both proceeded to TKA. At the time of study close, 29 patients (70.7%) in the LAGB group had undergone TKA and 12 had been removed from the waiting list because of symptom improvement. Of the 41 participants allocated to TAU, at the time of study close, 39 (95.1%) had undergone TKA, and 2 were removed from the waiting list because of worsening health issues and change of mind after obtaining a second opinion. All randomized participants were monitored from study enrollment to study closure.

**Table 1.** Baseline Characteristics of Participants, by Group

Characteristic	Participants, No. (%)	
	TAU (n = 41)	LAGB (n = 41)
Age, mean (SD), y	57.0 (5.7)	58.7 (3.7)
Sex		
Female	34 (82.9)	32 (78.0)
Male	7 (17.1)	9 (22.0)
Socioeconomic status score, mean (SD)	5.4 (2.7)	5.6 (2.6)
Charlson Comorbidity Index score		
0	21 (51.2)	19 (46.3)
1	12 (29.3)	16 (39.0)
≥2	8 (19.5)	6 (14.6)
Hypertension	26 (63.4)	27 (35.9)
Dyslipidemia	12 (29.3)	11 (26.8)
Diabetes	11 (26.8)	13 (31.7)
Smoking status		
Current	9 (22.0)	6 (14.6)
Former	11 (26.8)	16 (39.0)
Never	21 (51.2)	19 (46.3)
Body mass index, mean (SD)^a^	43.6 (6.3)	43.8 (4.8)
Weight, mean (SD), kg	114.0 (15.4)	116.1 (18.0)
Waist-to-hip ratio, mean (SD)	0.9 (0.2)	0.9 (0.1)
Western Ontario McMaster Universities Osteoarthritis Index score, mean (SD)^b^		
Pain	67.9 (14.2)	60.4 (12.7)
Function	65.5 (16.7)	59.8 (14.1)
Stiffness	69.8 (21.8)	67.7 (15.0)
Global	64.6 (17.2)	60.6 (12.8)
Veterans Rand 12 item score, mean (SD)^c^		
Physical component	25.6 (9.0)	23.8 (6.0)
Mental component	41.2 (14.2)	43.7 (16.2)

Abbreviations: LAGB, laparoscopic adjustable gastric banding; TAU, treatment as usual.

^a^ Body mass index is calculated as weight in kilograms divided by height in meters squared.

^b^ Scores ranged from 0 to 100, with higher scores denoting worse pain and greater disability. Actual ranges for individual scores in this study are as follows: pain (TAU, 35.0-100.0; LAGB, 25.0-85.0), function (TAU, 29.4-100.0; LAGB, 26.5-91.2), stiffness (TAU, 25.0-100.0; LAGB, 37.5-100.0), and global (TAU, 16.7-100.0; LAGB, 27.1-86.5).

^c^ A higher score denotes better health-related quality of life. There are no official upper and lower limits; rather, scores are compared with the population average. The population average for both the physical and mental components is 50.0. Actual ranges for individual scores in our study are as follows: physical (TAU, 9.9-47.3; LAGB, 8.7-36.3) and mental (TAU, 15.9-68.9; LAGB, 12.1-71.0).

### Timing of the Surgical Procedures

Of the 39 patients in the intervention group who underwent LAGB, the median (IQR) time from randomization to LAGB was 64 (48-90) days, and of the 29 patients who subsequently proceeded to TKA, the median (IQR) time from randomization to TKA was 522 (341-824) days, or 17 (11-25) months. Of the 39 patients in the TAU group who underwent TKA, the median (IQR) time from randomization to TKA was 118 (52-213) days or 4 (2-7) months (eFigure in Supplement 2).

### Primary Outcome

**Figure 2.**
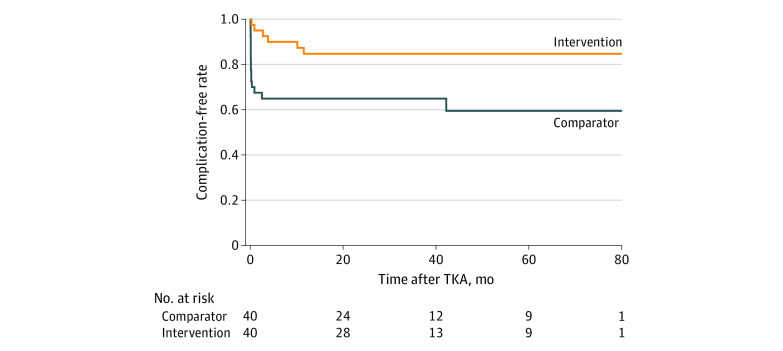
Time to Event Analysis TKA indicates total knee arthroplasty.

Six patients (14.6%) in the intervention group incurred the primary outcome, compared with 15 patients (36.6%) in the TAU group (difference, 22.0%; 95% CI, 3.7%-40.3%; *P* = .02) (Table 2). In the intervention group, all complications were single occurrences, whereas in the TAU group, 5 participants incurred multiple complications (20 events in 15 patients). The longest time after TKA when a primary outcome occurred was 10.1 months in the intervention group and 2.5 months in the TAU group. The Kaplan-Meier curves for the time to the first occurrence of a primary outcome are displayed in Figure 2. The hazard ratio was 0.29 (95% CI, 0.10-0.80; *P* = .02).

**Table 2.** Complications After Total Knee Arthroplasty, by Group

Outcome	Participants, No.	
	Treatment as usual (n = 41)	Laparoscopic adjustable gastric banding (n = 41)
Deep venous thrombosis^a^	1	1
Arrythmia^a^	1	1
Delirium^a^	3	0
Deranged liver function tests^a^	1	0
Nausea and vomiting^a^	1	0
Chest pain^a^	1	0
Bacteremia^a^	1	0
Wound complication^a^	8	1
Periprosthetic fracture^b^	0	1
Knee stiffness^b^	3	1
Loose screw^b^	0	1
Total		
Participants with complication(s)	15	6
No. of complications	20	6
Reoperation	2	3
Readmission	5	3
Revision^c^	1	0
Death^c^	1	0

^a^ Resulted in additional treatment and/or delayed discharge.

^b^ Required additional surgery.

^c^ Occurred more than 12 months after total knee arthroplasty.

In terms of the follow-up period for the primary outcome, among all patients who underwent TKA, time was counted from the day of the TKA. Among the 12 patients in the intervention group who did not undergo TKA, time was counted from 522 days after randomization, which was the median number of days from randomization to TKA among the 29 patients who underwent TKA. Among the 2 patients in the TAU group who did not undergo TKA, time was counted from 118 days after randomization, which was the median number of days from randomization to TKA among the 39 patients who underwent TKA. Participants who did not undergo TKA obviously did not experience any primary outcomes but contributed to person-time of follow-up according to an intention-to-treat principle. On the basis of the aforementioned assumptions, the median (IQR) follow-up after TKA was 24 (0-43) months for the intervention group and 27 (12-52) months for the TAU group (eFigure in Supplement 2).

### Secondary Outcomes

Among patients who underwent TKA, no difference was observed in the mean (SD) length of hospital stay between the intervention (4.4 [1.2] days) and TAU (5.0 [2.6] days) groups. Five patients (17.2%) required inpatient rehabilitation in the intervention group, compared with 4 patients (10.3%) in the TAU group (Table 3). For change in BMI, weight, and patient-reported outcomes, 12 months were counted from the day of TKA. For patients in the intervention group who did not undergo TKA, 24 months after bariatric surgery were counted as the 12-month post-TKA time point. This allowed for the a priori established maximum time to lose 20% of baseline body weight after LAGB and then 12 months of follow-up. No between-group differences in pain, function, or QoL were observed. The between-group difference in the mean BMI was −6.32 (95% CI, –7.90 to –4.50; *P* < .001) and that for mean weight was −16.5 kg (95% CI, –21.0 to –12.0 kg; *P* < .001).

**Table 3.** Scores on Continuous Outcome Measures, by Group

Outcomes	Mean (SD)		Difference in outcome between TAU and LAGB groups, mean (95% CI)
	TAU (n = 41)	LAGB (n = 41)	
Body mass index^a^			
Baseline	43.6 (6.3)	43.8 (4.8)	–6.32 (–7.90 to –4.50)^b^
12 mo	42.5 (6.6)	36.5 (5.5)	
Weight, kg			
Baseline	114.0 (15.4)	116.1 (18.0)	–16.5 (–21.0 to –12.0)^b^
12 mo	111.5 (17.0)	96.6 (17.1)	
Western Ontario and McMaster Universities Osteoarthritis Index score^c^			
Pain			
Baseline	67.9 (14.2)	60.4 (12.7)	0.6 (–9.6 to 10.9)
12 mo	23.4 (23.6)	21.1 (22.2)	
Function			
Baseline	65.5 (16.7)	59.8 (14.1)	–4.7 (–12.6 to 3.1)
12 mo	27.5 (18.6)	20.8 (18.0)	
Stiffness			
Baseline	69.8 (21.8)	67.8 (15.0)	–6.5 (–16.1 to 3.1)
12 mo	34.5 (24.0)	27.4 (20.2)	
Global			
Baseline	64.6 (17.2)	60.6 (12.8)	–5.0 (–13.1 to 3.1)
12 mo	27.3 (19.1)	21.4 (17.7)	
Veterans Rand 12 item Health Questionnaire score^d^			
Physical component			
Baseline	25.6 (9.0)	23.8 (6.0)	3.8 (–0.8 to 8.6)
12 mo	37.0 (11.6)	40.3 (9.8)	
Mental component			
Baseline	41.2 (14.2)	43.7 (16.2)	4.0 (–1.4 to 9.4)
12 mo	48.8 (12.1)	53.2 (12.6)	

Abbreviations: LAGB, laparoscopic adjustable gastric banding; TAU, treatment as usual.

^a^ Body mass index is calculated as weight in kilograms divided by height in meters squared.

^b^ *P* < .001.

^c^ Scores ranged from 0 to 100, with higher scores denoting worse pain and greater disability. Actual baseline ranges for individual scores in this study are as follows: pain (TAU, 35.0-100.0; LAGB, 25.0-85.0), function (TAU, 29.4-100.0; LAGB, 26.5-91.2), stiffness (TAU, 25.0-100.0; LAGB, 37.5-100.0), and global (TAU, 16.7-100.0; LAGB, 27.1-86.5). Actual ranges for 12 months are as follows: pain (TAU, 0.0-95.0; LAGB, 0-75), function (TAU, 1.5-75.1; LAGB, 0.0-61.8), stiffness (TAU, 0.0-75.0; LAGB, 0.0-75.0), and global (TAU, 3.1-71.9; LAGB, 0.0-71.8).

^d^ A higher score denotes better health-related quality of life. There are no official upper and lower limits; rather, scores are compared with the population average. The population average for both the physical and mental components is 50.0. Actual ranges at baseline for individual scores in our study are as follows: physical (TAU, 9.9-47.3; LAGB, 8.7-36.3) and mental (TAU, 15.9-68.9; LAGB, 12.1-71.0). Actual ranges at 12 months are as follows: physical (TAU, 12.7-58.1; LAGB, 19.7-56.8) and mental (TAU, 20.6-66.0; LAGB, 21.7-68.1).

### Adverse Events

Two patients experienced post-LAGB infective complications, resulting in removal of the band and port without subsequent replacement. One participant developed a port incision infection, requiring revision of the port only. These patients progressed to TKA, and 2 subsequently incurred the primary outcome. At the time of study closeout, 1 participant had undergone revision TKA at 3 years because of injury following a fall, and there was 1 death at 6 years unrelated to TKA. Both patients were in the TAU group.

## Discussion

In this assessor-blinded RCT, among patients with severe obesity and knee OA who underwent TKA, significantly fewer patients who underwent bariatric surgery experienced a post-TKA complication compared with those who underwent TKA alone. The main factor associated with this difference was the group of participants (30.8%) who declined to proceed with TKA because of symptom improvement with weight loss following their bariatric procedure. This was despite participants meeting clinical and radiographic criteria for TKA^29^ on initial clinical screening.

To our knowledge, this is the first RCT to assess the efficacy of substantial weight loss induced by bariatric surgery in reducing complication risk after TKA in patients with severe obesity and knee OA. In previous uncontrolled studies^17,30^ of bariatric surgery before TKA, findings have been equivocal. Lower risk of complications and death have been recorded in patients who had undergone bariatric surgery before TKA,^31^ and yet the inverse has also been reported.^30^ Possible explanations for the inconsistent findings include inaccuracies with retrospectively reviewing medical records^32^ and incomplete data capture arising from short-term follow-up.^33^

A striking finding of our study was that nearly one-third of patients had not proceeded with planned TKA up to 5 years after bariatric surgery. Although not the primary intent of our study, these results indicate that for a substantial portion of patients with severe obesity and knee OA, symptoms may be effectively managed with weight-loss strategies alone. This is supported by a prior smaller study^5^ that demonstrated a positive association between BMI change and pain and functional improvement 6 months after bariatric surgery in patients with severe obesity and knee OA, with patients inclined to defer TKA.

The weight loss achieved in the intervention group was substantial and clinically significant (weight 16 kg or BMI 6.32 sustained at 12 months after TKA) and was very closely associated with the improved outcomes observed. Newer bariatric surgical modalities such as sleeve gastrectomy typically induce greater weight loss accompanied by improved patient satisfaction when compared with LAGB.^23^ It is, therefore, probable that these same positive outcomes will be achieved with other bariatric surgical procedures before TKA. Weight loss similar to that seen in our study, for more than 1 year, has recently been demonstrated in a trial using a medical therapy.^34^ This provides the opportunity to avoid another surgical procedure before TKA, with the caveat that longer-term outcomes are uncertain, as are the effects in patients with severe obesity.

We experienced several unanticipated but important challenges with the conduct of this study, which must be considered when interpreting our findings. LAGB surgery itself was not without risk, with 3 infective complications occurring in this group. This complication rate is higher than has been noted on the Australian and New Zealand Bariatric Surgery Registry (1.3%),^35^ which may be reflective of more diligent outcome reporting in a controlled trial, the older age of the cohort, and the comorbidities of the patients. Our reported complication rate was similar to that reported in a recent study examining bariatric surgery in patients aged 65 years and older.^36^ The greater challenge occurred because of patients deferring TKA surgery after weight loss, thus substantially prolonging the study timelines. Although progression to TKA after weight loss was not the primary intent of our study, this scenario warrants further investigation and conduct of future trials of surgical weight loss with progression to TKA surgery as the primary outcome.

### Limitations

Our study has limitations in terms of its generalizability. Although a significant reduction in complication risk was demonstrated after weight loss surgery, there is currently no consensus regarding the age limit for bariatric surgery in older adults. Our study imposed a 65-year age limit, which was the upper age limit for bariatric surgery in our Victorian public health system at the time. Since commencement of our trial, several reports^37,38,39^ favoring surgery in patients older than 60 or 70 years have become available. A recent analysis of 2 years of US Medicare data identified 8510 patients older than 65 years undergoing bariatric surgery and concluded it was a safe and effective intervention.^40^ Patients considering TKA may benefit substantially from bariatric surgery in terms of improved metabolic comorbidities, such as insulin resistance and type 2 diabetes, as well as improved overall QoL.^41^ Although bariatric surgery may not be implemented routinely, or deemed acceptable by all patients before TKA, achieving substantial weight loss should be strongly considered as a means of improving outcomes in patients with severe obesity.

## Conclusions

The findings of this RCT suggest that weight loss before TKA reduces the risk of complications after TKA in patients with BMI greater than 35 and OA. It also appears to result in a substantial proportion of patients deferring TKA surgery.

## Notes

Supplement 1.
Trial Protocol and Statistical Analysis Plan

Supplement 2. **eTable.** Reason for Study Exclusion **eFigure.** Study Timelines

Supplement 3.
Data Sharing Statement
