# Supplementary material for: Effect of Bariatric Surgery on Risk of Complications After Total Knee Arthroplasty: A Randomized Clinical Trial
Source: JAMA Netw Open. 2022 Apr 14;5(4):e226722. doi: 10.1001/jamanetworkopen.2022.6722 (PMC9011119; doi:10.1001/jamanetworkopen.2022.6722)
Supplement: Supplement 1. — Trial Protocol and Statistical Analysis Plan [file jamanetwopen-e226722-s001.pdf]

Clinical Research Protocol

ABS Study

The Arthroplasty and Bariatric Surgery (ABS) study: a randomised controlled trial of laparoscopic adjustable gastric banding prior to total knee arthroplasty.

Study Protocol

Version 1.0

This study is registered on Australian New Zealand Clinical Trials Registry (ANZCTR)
Identification no.: 12611001178932

Principal Investigator: Professor Peter Choong, MBBS, MD FRACS, FAOrthA, FAAHMS, MAICD
Sir Hugh Devine Professor of Surgery
Head of Department of Surgery Melbourne | Medical School
Faculty Medicine, Dentistry & Health Sciences
University of Melbourne

Chair, Bone and Soft Tissue Tumour Unit
St Vincent's Hospital Melbourne | Peter MacCallum Cancer Centre

University: University of Melbourne
Department of Surgery | St Vincent's Hospital Melbourne
Levels 2 Clinical Sciences Building
29 Regent Street, Fitzroy 3065, Victoria. Australia

Funding Sponsors: National Health and Medical Council (NHMRC)

Study Protocol

Version 1.0 19th January 2012

Approved by St Vincent's Hospital HREC-A

Reference No. 142 / 11

Investigators

Chief Investigator A: Professor Peter Choong
Organisation: University of Melbourne and St. Vincent's Hospital
Address: PO Box 2900, Fitzroy VIC 3065
Telephone: +61392312365
Email: pchoong@unimelb.edu.au

Chief Investigator B: A/Prof Wendy Brown
Organisation: Monash University and The Alfred Centre
Address: 99 Commercial Rd, Melbourne VIC 3004
Telephone: +61399030725
Email: wendy.brown@monash.edu

Chief Investigator C: Professor Danny Liew
Organisation: Monash University and School of population Health and Preventative Medicine
Address: 533 St Kilda Rd, Melbourne VIC 3004
Telephone: +61399030759
Email: danny.liew@monash.edu

Chief Investigator D: A/Prof Michelle Dowsey
Organization: University of Melbourne and St. Vincent's Hospital
Address: PO Box 2900, Fitzroy VIC 3065
Telephone: +61392313955
Email: mmdowsey@unimelb.edu.au

Associate Investigator: Emeritus Professor Paul O'Brien
Organisation: Monash University and The Alfred Centre
Address: 99 Commercial Rd, Melbourne VIC 3004
Telephone: +61399030725
Email: paul.obrien@monash.edu

Study Co-ordinator: Angela Cochrane
Organization: St. Vincent's Hospital Melbourne
Address: PO Box 2900, Fitzroy VIC 3065
Telephone: +61392312364
Email: angela.cochrane@svha.org.au

Table of Contents

Investigators 2

List of Abbreviations 5

Study Summary 6

1.0 Introduction 7

1.1 Impact of obesity..7

1.2 Obesity and Clinical Outcomes..7

1.3 Obesity and Economic Outcomes..8

1.4 Obesity and implant failures..8

1.5 Obesity and functional Quality of Life Outcomes 8

1.6 Summary of Background and Rationale..9

1.7 Obesity, Weight Loss and Bariatric Surgery 10

1.8 Scope of Presenting Issue..10

1.9 Joint Arthroplasty and Bariatric Surgery 10

1.10 Dieting and Pre joint Arthroplasty 11

2.0 Study Objectives 11

2.1 Hypothesis 11

2.2 Definitive Research Objectives..12

2.2.1 Definitive Primary Research Objectives..12

2.2.2 Definitive Secondary Research Objectives..12

3.0 Study Design 12

3.1 Study Design Overview 12

3.2 Outcome Measures 12

3.2.1 Primary Study Endpoints 12

3.2.2 Secondary Study Endpoints 13

4.0 Study Methods and Procedures 13

4.1 Study Setting 13

4.2 Patient Eligibility Criteria 14

4.2.1 Inclusion Criteria 14

4.2.2 Exclusion Criteria 14

4.3 Recruitment Strategy and Patient Screening 14

4.4 Consent Process 14

4.5 Randomisation 15

4.6 Study Intervention 15
4.6.1 Intervention Group: LAGB + TKA 15
4.6.2 Control Group: TKA Alone 16
4.7 Participant Follow-Up 16
4.7.1 Intervention Group (LAGB + TKA) Follow-Up 16
4.7.2 Control Group (TKA Alone) Follow-Up 16
4.8 Protection against sources of bias 16
5.0 Statistical Plan 17
5.1 Sample Size Determination 17
5.2 Statistical Analysis Plan 17
5.2.1 Health Economic Analysis....17
5.2.2 Analysis of Feasibility of Outcomes....17
6.0 Data Collection and Management....18
6.1 Data Collection 18
6.1.1 Patient randomised to intervention group (LAGB + TKA) 18
6.1.2 Patient randomised to control group (TKA alone)....19
6.2 Data Integrity 20
7.0 Ethical Considerations 20
7.1 Research Ethics Approval 20
7.2 Informed Consent 20
7.3 Confidentiality 20
7.4 Protocol Amendment 21
7.5 Safety and Adverse Events 21
7.5.1 Clinical Site Notification....21
7.5.1.1 Notifying St Vincent's Hospital 21
7.5.1.2 Notifying the Appropriate Ethics Committee 21
8.0 Clinical Impact and Knowledge Dissemination 21
8.1 Potential Clinical Impact of the Study....21
8.2 Realisation of Clinical Impact 21
8.3 Importance of Health Economic Modelling 22
8.4 Building Translational Research Capacity....22
8.5 High Impact Publications 22
9.0 Study Time Line....22
10.0 References....23

List of Abbreviations

ABS	Arthroplasty and Bariatric Surgery study
AE	Adverse Event
AI	Associate Investigator Professor Paul O'brien
BMI	Body Mass Index
CBS	The Centre for Bariatric Surgery
CIA	Chief Investigator Professor Choong
CIB	Chief Investigator A/Prof Brown
CIC	Chief Investigator Prof Liew
CID	Chief Investigator A/Prof Dowsey
CORE	Centre of Obesity Research and Education
CVA	Cerebrovascular accident
EWL	Excess Weight Loss
GCP	Good Clinical Practice
IKS	Knee Society Score
LAGB	Laparoscopic Adjustable Gastric Banding
NHMRC	National Health and Medical Research Council
OA	Osteoarthritis
PJI	Prosthetic Joint Infection
QALY	Quality-adjusted life years
QoL	Quality of Life
SAE	Serious Adverse Event
SF12	Short Form 12
SVHM	St Vincent's Hospital Melbourne
TJA	Total Joint Arthroplasty
TKA	Total Knee Arthroplasty
VTE	Venous Thromboembolism

Study Summary

**Title**	The Arthroplasty and Bariatric Surgery (ABS) study: a randomised controlled trial of laparoscopic adjustable gastric banding prior to total knee arthroplasty.
**Short Title**	ABS Study
**Methodology**	Multi Centre Prospective Randomised Controlled trial
**Coordinating Centre**	St Vincent's Public Hospital Melbourne
**Clinical Sites**	St Vincent's Public Hospital Melbourne, The Avenue Private Hospital and The Alfred Hospital
**Primary Objective**	Our overall objective is to determine whether clinical, functional and quality of life (QoL) outcomes in severely obese patients (with body mass indices [BMIs] of ≥ 35 kg/m^2) undergoing total knee arthroplasty (TKA) will be improved if this is preceded by laparoscopic adjustable gastric banding (LAGB)
**Secondary Objective**	To undertake a modelled health economic analysis of LAGB+TKA versus TKA alone in severely obese patients.
**Treatment Groups**	The study will compare an intervention group (LAGB + TKA) to a control group (TKA alone) in severely obese (BMI ≥35 kg/m^2) patients with end stage OA
**Study Outcome**	Outcomes will be compared at 12 months after TKA
**Follow-Up**	For the Intervention group (LAGB + TKA) participants will be referred back to SVHM 12 months post LAGB or after 20% loss of baseline body weight if this occurs earlier for review with the Orthopaedic Surgeon.<br>For the Control group (TKA alone) participants will undergo TKA with routine follow-up care
**Sample Size**	Total 120 participants in each of the 2 (equally sized) groups
**Estimated Study Duration**	4 years is estimated, 2 years allocated to recruitment, 12 month follow-up after LAGB and 12 month follow-up after TKA
**Significance**	This project targets obesity and arthritis, two health priorities in this country. TKA, a high volume and already expensive procedure is being threatened by the increasing epidemic of obesity, which stands to multiply post-operative complications and escalate disproportionately the cost of OA care. It is important to note that obesity is over-represented by almost 3:1 in patients presenting for TKA versus the community. There is an urgent need to identify patient and treatment parameters that may mitigate these complications and provide for better outcomes. Potential savings from reducing infection alone are conservatively assessed at $66 million per year. Health economic modelling of the proposed interventional strategy has not been undertaken before, yet is critical to informing health policy.

1.0 Introduction

1.1 Impact of Obesity

Obesity is so detrimental to public health that it is now regarded a global health priority (1, 2). The causes of obesity are multi-factorial, but essentially arise from caloric intake that exceeds energy expenditure. While this equation is simple, achieving weight control and preventing obesity remain elusive and the prevalence of obesity in Australia continues to rise (1). Understanding the causes of obesity is important for its prevention, but more urgent is the need to examine the efficacy of current obesity treatments and how these may impact coexistent conditions.

OA is common and increases in prevalence with age. The extent of the health and financial burden on the Australian community arising from OA is so significant that the Australian government has joined with the World Health Organisation to designate this era as the “Bone and Joint Decade”. The commonest cause of OA is age-related “wear and tear”, while other less common but still important causes of OA include post-trauma, inflammatory joint disease, excessive alcohol consumption and steroid use(3). These latter causes account for fewer than 10% of all total joint arthroplasties (TJA)(3).

The association between OA and obesity (4) is clear and the economic impact of obesity-related OA in Australia was estimated to be $221.3 million in 2005 alone (5). Contributing to this burden is the fact that obese patients are more likely to require TJA (6) and short and long term outcomes post-surgery are known to be inferior compared to non-obese patients (7-13). It is not clear if there are potential benefits for patients if they have their obesity treated prior to TJA. This is of critical importance because over 80,000 patients undergo TJA in Australia each year, and a significant proportion are obese (14, 15).

St. Vincent’s Hospital Melbourne (SVHM) is a tertiary referral centre for joint replacement surgery. The hospital’s Department of Orthopaedics is a leading academic adult orthopaedic centre in Australia, with a major focus on joint arthroplasty. The Victorian Department of Human Services has designated SVHM a Centre of Excellence for joint arthroplasty and currently over 750 TJAs are performed at this site annually. This represents one of the largest such services in Australia. The SVHM Department of Orthopaedics pursues a major research interest in predictors of outcomes after TJA and maintains a comprehensive database of all lower limb primary arthroplasties performed since 1998. Extensive follow-up data to 12 years are also captured. In effect, we are maintaining a large prospective cohort study of patients undergoing TJA at SVHM and now have a consecutive series of more than 7000 procedures, involving 6000 patients.

From our data emerges the fact that obesity is common among patients undergoing TKA (15). Indeed, 60% of the 3700 knee procedures recorded to date have been performed on obese patients. We have therefore launched a series of studies to determine the extent to which obesity influences outcomes post TKA. This includes a major prospective study investigating the prognostic significance of obesity on joint arthroplasty (NHMRC Scholarship #502021), with the specific aims of (a) determining if lower limb arthroplasty influences weight change in obese patients, (b) determining the risk of complications associated with obesity and (c) comparing function and quality of life outcomes between obese and non-obese groups (14, 15).

1.2 Obesity and Clinical Outcomes

In a series of 1214 patients, we reported that the odds of developing a prosthetic joint infection (PJI) in morbidly obese (BMI ≥ 40kg/m2) individuals undergoing TKA was 9 times that of their non- morbidly obese counterparts, and that any level of obesity combined with diabetes increased the likelihood of developing a

PJI (16). Others have also reported that obese and severely obese patients who undergo TKA are at significant risk of a number of early complications, including a higher incidence of multiple complications and infection, prolonged wound drainage, prolonged length of stay, higher costs for inpatient rehabilitation, avulsion of the medial collateral ligament and a higher risk of thromboembolic events (8-11, 13). Consequently, many authors advocate counselling obese patients to lose weight prior to TJA. However, even the most intensive non-surgical weight loss programs, including new pharmacological therapies, have reported only modest success in the short term and failure to sustain weight loss in the longer term(17), (18, 19). Obese patients with end-stage OA awaiting TKA have the added disadvantage of a significant functional disability and pain, compounding their ability to participate effectively in most activity-related weight loss programs.

1.3 Obesity and Economic Outcomes

The cost of TKA is significantly higher in obese patients compared to non-obese patients (20). In a recent study involving 530 consecutive TKA's, we found that the mean cost per episode of care was $1,821 more in obese compared to non-obese patients, even after adjusting for; age, gender, aetiology, cardiovascular disease, diabetes and smoking (article in press). These represent in-patient costs only and hence are likely to have underestimated the true difference in TKA costs between obese and non-obese patients.

1.4 Obesity and Implant Failure

Obesity has been shown to exert a profound impact on tibial component failure if coexistent with varus tibial alignment. A large study of 3152 TKAs reported a 168-fold increase in component failure when BMI exceeded 33.7 kg/m² (21). The investigators reported that failure at this level of obesity was also associated with a varus tibial alignment greater than 3 degrees. In this regard, we have demonstrated in a number of studies, including a recent randomised trial, that obesity was responsible for greater inaccuracy of implantation (22, 23) and that coronal alignment within 3 degrees of neutral was only achieved in 56% of obese patients who underwent conventional TKA (23), thus potentially exposing them to the negative effect of obesity on component mal-alignment. However, merely making efforts to avoid mal-alignment of knee prostheses in obese patients may not resolve this issue. Several recent studies draw similarities in the gait pattern of obese patients with that seen in OA, and point to excess loading as a potential precursor of joint wear (24, 25). In two studies, the obese group walked slower and had a shorter stride length, and spent more time on stance phase and double support in walking. Greater hip adduction was shown in the obese group during terminal stance and pre-swing. The maximum knee adduction angles of the obese group in both stance and swing phases were significantly higher. In a third study, weight alone explained the peak external adduction moment during level gait that drives excessive medial pressures on the knee (26). A recent radiostereophotometric analysis of knee prosthesis function mapped the gradual subsidence of knee prostheses within the tibia over a year after TKA in obese patients highlighting the effect of the abnormal gait of obese patients on their knee implants (27). It is clear from these studies that unless obesity is corrected and weight loss maintained, the prosthetic knee would be exposed to the same biomechanics that led to joint degeneration in the first place, thus making the implanted prosthesis vulnerable to accelerated wear and failure.

1.5 Obesity and Functional QoL Outcomes

In a recent literature review, we reported the paucity of research into the functional and quality of life (QoL) outcomes for obese patients undergoing TKA (28). While functional and QoL improvements for all weight groups are reported following TKA, there is generally a lesser degree of improvement in obese patients compared to non-obese patients (13, 29).

1.6 Summary of Background and Rationale

Our research initiative (NHMRC Scholarship #502021) examined the prognostic significance of obesity in joint arthroplasty involving clinical, functional and QoL outcome studies on more than 1100 consecutive patients who have undergone TJA at SVHM since 2006, 597 of these being TKA. This is the largest study of its kind to report on weight change following TKA. Among the 597 patients who underwent TKA, 62% were obese or worse. (Table 1).

Table 1. BMI distribution of 597 patients who underwent TKA and weight status at 12 months.

Weight Category (BMI kg/m2)	Number pre Surgery (%)	Number post-surgery (%)	Number who lost ≥10% weight (%)	Number who gained ≥5% weight (%)
Underweight (<19)	3 (1%)	3 (1%)	0 (0%)	1 (33%)
Normal weight (19-25)	62 (10%)	54 (9%)	1 (2%)	19 (30%)
Over weight (25-29)	162 (27%)	157 (27%)	6 (4%)	29 (18%)
Obese (30-34)	184 (31%)	171 (29%)	4 (2%)	38 (21%)
Severely obese (35-39)	125 (21%)	130 (22%)	4 (3%)	20 (16%)
Morbidly obese (≥40)	61 (10%)	73 (12%)	1 (2%)	7 (11%)
Total	597	588	16 (3%)	114 (19%)

Activity-related weight loss in obese patients awaiting TJA is a problem because the symptoms of disabling arthritis may limit an individual's ability to exercise. Patients often identify this as the reason for the inability to lose weight, and believe that joint replacement would be critical for weight loss. While QoL surveys demonstrate that significant improvements are achieved in pain and function for all weight groups following joint replacement (7), our results show that less than 3% of patients in the obese groups (n=9) lost 10% or more of weight 12 months following TKA (Table 1). Morbidly obese patients showed the least weight loss. A 10% weight reduction in overweight or obese individuals is important because this correlates with significant improvements in cardiovascular and metabolic health (30). Of note, and contrary to popular patient belief, 19% of the total patient group *gained* 5% or more of their original body weight 12 months after TKA.

We have also demonstrated that increasing weight correlated with poorer function and QoL scores, and that the degree of improvement after surgery in terms of function and QoL were inversely related to BMI (Table 2). Of particular concern was the 20% *increase* in the number of morbidly obese patients at completion of follow-up compared to pre surgery.

Table 2. Function and quality of life score pre and 12 months post total knee arthroplasty.

	Normal Weight	Overweight	Obese	Severely Obese	Morbidly Obese	P
Pre IKS	77.1 + 25.5	77.0 + 23.7	70.9 + 24.6	67.5 + 24.0	64.7 + 24.8	0.042
12m IKS	148.7 + 32.7	141.9 + 35.0	134.8 + 36.6	127.5 + 37.6	123.0 + 33.8	<0.001
Pre QoL	27.4 + 6.2	26.33 + 6.0	25.69 + 5.8	25.82 + 5.1	24.63 + 3.7	0.060
12m QoL	40.5 + 10.7	37.62 + 10.2	37.45 + 10.9	36.35 + 10.1	34.65 + 11.2	0.033

- Scores expressed in mean and standard deviation
- Function assessed using the Knee Society Score (IKS), pre and 12 months post-surgery
- Quality of life assessed using the Short Form 12 (SF12), pre and 12 months post-surgery

In addition to weight gain and poorer function and QoL outcomes, we found that the risk of incurring an

adverse event in the first 12 months after TKA was significantly higher in obese (23%) and morbidly obese (35%) patients, compared to non-obese patients (14%),(15). This also resulted in more unplanned readmissions, with 15% of obese patients and 23% of morbidly obese patients requiring readmission due to complications, compared to 5% of non-obese patients (15).

1.7 Obesity, Weight Loss and Bariatric Surgery

A recent Cochrane review established that surgical treatment of obesity is more effective for weight loss than conservative management, with surgically treated patients sustaining significant weight loss at 8 years (31). We have also reported that lifestyle interventions and pharmacological therapy appear to be ineffective for sustained weight loss in obese patients (18). LAGB has been shown in randomised trials to be more effective than optimal medical programs for achieving weight loss, health improvement and increased QoL, with no increase in the incidence of adverse events (19). We have also reported that LAGB is equally effective (19) and much safer (32) than other more invasive surgical techniques. LAGB results in 50-60% excess weight loss (EWL). Importantly, this effect appears to be durable (18) and is associated with major improvements in obesity-related co-morbidities such as diabetes, hypertension, reflux oesophagitis, asthma, depression, non-alcoholic steatatohepatitis, polycystic ovarian syndrome and sleep apnoea (31, 33-40). We recently confirmed the beneficial effect of LAGB on type 2 diabetes via a randomised controlled trial (41).

1.8 Scope of Presenting Issue

In 2010, nearly 40,000 knee replacements were recorded in Australia, representing an increase of 26% compared to 5 years previously (3). Based on extrapolation of the proportional distribution of weight categories among SVHM TKA patients (Table 1), approximately 25,000 Australians presenting for knee replacement per year would be obese. Of these nearly 8500 would be severely obese (BMI 35-39 kg/m2) and approximately 4000 morbidly obese (BMI ≥40 kg/m2). All these patients would be at risk of significant, yet highly preventable, complications from their obesity. Our results of a 9-fold increase in deep infection rates alone in morbidly obese patients presenting for TKA suggests that almost 400 out of the 4400 potentially morbidly obese patients in Australia undergoing TKA will develop peri-prosthetic infections each year. Recent estimates of the total inpatient treatment and outpatient follow-up costs for revision knee surgery resulting from prosthetic knee infection approaches $165,000 per patient (42). Thus the total financial burden each year could be estimated as high as $66 million for obesity related infection alone. This is likely to be an underestimate of the overall cost as it does not include the many other non-septic obesity related co-morbidities that require treatment.

1.9 Joint Arthroplasty and Bariatric Surgery

Given the high risk of poor outcomes and the economic burden of treating these in obese patients undergoing TKA, denying surgery for obese patients who require TKA may seem a valid approach. However, the likely outcome for these patients is continued functional decline, worsening health and loss of independence, thus creating an even greater demand on patients, their carers and the community. Bariatric surgery may provide a clinically and economically viable solution for pre-operative weight loss in obese patients. With its current track record as a safe, technically established, reproducible and effective weight loss option when performed routinely in specialist centres, LAGB represents an ideal modality for testing the clinical significance of weight reduction in obese osteoarthritic patients requiring TKA. There is a dearth of literature regarding the effects of bariatric surgery in joint replacement other than a limited uncontrolled review of 20 morbidly obese patients who underwent TJA after bariatric surgery (43). This report suggested a positive outcome for these

patients. In comparison to diet-only programmes, LAGB appears attractive for mitigating the consequences of obesity on knee implants described above because of its established ability to maintain weight reduction in the medium to long term.

1.10 Dietary Intervention and Pre-Joint Arthroplasty

Weight loss through dietary intervention in obese patients with OA has been trialled with only moderate success (44-46). Two separate trials examining weight loss programs in obese patients with knee OA, reported losses between 4.9% (dietary program) and 8.7% (intensive weight loss program) of baseline weight over 6 to 18 months (45, 46). However, in both studies, the average BMI of participants was <35kg/m2. Our project targets severely obese patients (BMI>35kgm/2), which accounts for 31% of patients undergoing TKA (NHMRC #502021). Applying 8.7% weight loss to the severely obese group would result in only 25% of patients reducing their BMI to less than 35kg/m2 and no patient would shift into a non-obese category (BMI<30kg/m2). Accordingly, even the most intensive pre-operative dietary program is unlikely to positively influence the high rate of complications incurred in severely obese patients undergoing TKA. LAGB prior to TKA may result in sufficient weight loss so as to reduce the risk of post-operative complications, improve joint function and mobility, and thus facilitate better health from reduced weight and improved exercise tolerance. It would be of considerable interest to know if these benefits are also cost effective. This information would be critical for developing safer, more effective and cost-effective programmes for TKA in obese patients.

2.0 Study Objectives

The overarching objective of this trial is to answer the following research question:

Will clinical, functional and quality of life (QoL) outcomes in severely obese patients (with body mass indices [BMIs] of ≥ 35 kg/m²) undergoing total knee arthroplasty (TKA) be improved if this is preceded by laparoscopic adjustable gastric banding (LAGB) ?

2.1 Hypothesis

The null hypothesis proposes that clinical, functional and quality of life (QoL) outcomes in severely obese patients (with body mass indices [BMIs] of ≥35 kg/m2) undergoing total knee arthroplasty **(**TKA) will not be improved if this is preceded by laparoscopic adjustable gastric banding (LAGB).

2.2 Definitive Research Objectives

2.2.1 Definitive Primary Research Objectives

a) To determine if clinical, functional and QoL outcomes are improved by combining LAGB with TKA (LAGB+TKA) in severely obese (BMIs ≥35 kg/m2) patients with end-stage osteoarthritis (OA) as compared to TKA alone.

2.2.2 Definitive Secondary Research Objectives

a) To undertake a modelled health economic analysis of LAGB+TKA versus TKA alone in severely obese patients.

3.0 Study Design

3.1 Study Design Overview

We propose to conduct a randomised controlled trial to compare LAGB+TKA with TKA alone in severely obese (BMI ≥ 35 kg/m2) patients with end-stage OA. Outcomes will be compared at 12 months after TKA. As a prospective randomised controlled trial, the study strategy will be constructed and presented according to the recommendations of the CONSORT statement (47).

3.2 Outcome measures

As part of the standard of care when undergoing TKA at SVHM, patients are routinely followed up at 6 weeks, 3 months and 12 months post-surgery. During these visits, routine follow-up includes capture of the outcomes mentioned below. Thereafter, follow-up occurs at 2, 5, 7 and 10 years.

3.2.1 Primary Study Endpoints

The primary outcome will be a composite of:

(i) death from any cause

(ii) peri-operative complications (medical or surgical)

(iii) post-operative complications (medical or surgical) that result in a delay in discharge, eg: fracture, neuropraxia, sepsis, nosocomial infection, myocardial infarction, bowel obstruction, VTE, CVA, renal failure

(iv) Wound complications (breakdown, infections, haematomas, dehiscence)

(v) Joint infections (Centres for Disease Control definitions applied)

(vi) Unplanned procedures and/or readmissions in the first 12 months following TKA.

The processes for identifying outcomes will be conducted by the Project Research Officer who will be blinded to the intervention allocation. Active surveillance for outcomes will comprise the following:

- Recording of data regarding patient demographics (sex, age and country of birth), co-morbidities, and anaesthetic and surgery details on the day of surgery.
- Attendance at all unit ward rounds to capture complications and readmissions
- Attendance at X-ray meetings to capture technical complications of surgery
- Review of all medical records on patient discharge and follow-up at each outpatients review
- Recording of alignment of all post-operative full-leg standing radiographs
- Phone calls to participants at 3-monthly intervals
- Retrieval and review of records from other healthcare institutions as required.

The Project Officer will collate information on all possible primary outcomes of interest for review by an outcomes verification panel comprising at least 4 independent clinicians blinded to the patients' intervention allocation. The panel will include at least 2 peri-operative physicians and 2 orthopaedic surgeons. Ascertainment of outcomes will be undertaken according to review against strict criteria (to be determined and endorsed by the panel).

3.2.2 Secondary Study Endpoints

a. **Bed day utilisation** Length of stay for the index surgery, in-patient rehabilitation and subsequent related admissions in the first 12 months.

b. **Anthropomorphic measurements** BMI and waist hip ratio measurements will be taken in the orthopaedic PAC at monthly intervals post LAGB, prior to and at 12 months post TKA.

c. **Function and QoL scores** The Knee Society score (50) and SF-12(51) are both validated assessment tools for measuring health related QoL after TKA. These questionnaires will be administered prior to both LAGB and TKA procedures and annually after TKA.

d. **Blood indices** Blood for full blood examination, urea and electrolytes, renal function, measures of glucose intolerance, iron studies, vitamin B12 and D, folate, parathyroid hormone and calcium will be collected prior to and after LAGB and as part of routine monitoring pre and post this procedure.

e. **Health services utilisation** Information regarding utilisation of health services, including medications and in- and outpatient services, will be captured by linkage of our database to administrative and clinical databases maintained by SVHM. The latter capture extensive information on *all* healthcare encounters occurring at SVHM's hospital network, including administered medications as well as fully-itemised costs of healthcare encounters.

f. **Radiology** As is standard for patients undergoing TKA at SVHM, routine x-rays will be performed within 2 weeks prior to surgery, post-operatively and at 12 months post-surgery. This includes full-leg weight bearing radiographs enabling assessment of prosthetic alignment.

4.0 Study Methods and Procedures

4.1 Study Setting

The study will be coordinated by the Orthopaedic Research Department at St Vincent's Public Hospital in Melbourne (SVHM). The SVHM Orthopaedics Department is a leading academic adult orthopaedic centre in Australia, with a major focus on joint arthroplasty. The Victorian Department of Human Services has designated SVHM a Centre of Excellence for joint arthroplasty and currently over 750 TJAs are performed at this site annually. This represents one of the largest such services in Australia. The SVHM Department of Orthopaedics pursues a major research interest in predictors of outcomes after TJA and maintains a comprehensive database of all lower limb primary arthroplasties performed since 1998. Extensive follow-up data to 12 years are also captured. In effect, we are maintaining a *large prospective cohort study* of patients undergoing TJA at SVHM and now have a *consecutive* series of more than 7000 procedures, involving 6000 patients.

4.2 Patient Eligibility Criteria

All eligible patients will be recruited from the Orthopaedic Clinics at St Vincent's Hospital Melbourne following being consented for a primary TKA.

4.2.1 Inclusion Criteria

(i) Age ≤ 65 years at time of LAGB
(ii) BMI ≥35 kg/m^2
(iii) On the surgical waiting list for primary TKA at SVHM
(iv) Willing and able to cooperate in a long-term weight management programme.

4.2.2 Exclusion Criteria

(i) Revision surgery or surgery for neoplastic disease
(ii) Inability to provide informed consent
(iii) A medical condition which in the opinion of the investigators makes the patient unsuitable for participation in the trial
(iv) Previous oesophagogastric surgery such as fundoplication
(v) Lack of acceptance of the randomisation process.

4.3 Recruitment Strategy and Patient Screening

All patients on the waiting list for primary TKA have attended the orthopaedic pre-admission clinic at SVHM as part of their routine care will be screened for eligibility. This multidisciplinary pre-admission and review clinic comprises an orthopaedic surgeon, resident medical officer, nursing and allied health staff. Following consultation with the orthopaedic surgeon, eligible subjects will be approached by the study coordinator. After initial screening, potentially suitable subjects will be given a plain English statement, which will detail the problem of obesity and the nature of the study.

4.4 Consent Process

The consent process will take place once either in the orthopaedic clinic or over the phone by delegated study personnel, once consent for a TKA has been signed with the orthopaedic surgeon. The process of obtaining informed consent for the study will be completed in accordance with NHMRC guidelines, by certified study personnel and the following processes will be adhered to:

- Discuss with suitable candidates the study in a manner that is understandable
- Allow the potential participant to ask any questions he or she may have
- Provide the potential participant with the Patient Information Consent Form
- Provide an opportunity to discuss potential participation with his / her family, friends or family physician
- Confirm that the potential participant has an understanding of the potential risks and benefits of participating in the study
- Confirm that the potential participant understands that participation in the study is completely voluntary and that by choosing not to participant in the study will not affect their planned care at St Vincent's Hospital
- As written consent is required, this will be obtained at the participant's next clinic visits, or at time agreeable to both the study participant and delegated study personnel
- Complete and obtain appropriate signatures on the Informed Consent form and collect information form the participant
- Provide the participant with copies of the signed information consent form
- Study personnel will obtain informed consent prior to randomisation.

4.5 Randomisation

Subjects will also be informed of the potential outcomes of randomisation; the possibility of being assigned to either the treatment arm or the control arm, as detailed below. A second visit with the study coordinator will be arranged within 2 weeks for willing subjects, at which point specific questions regarding the trial will be answered and suitability for the study re-assessed. Study consent will occur at this point for suitable subjects willing to proceed. Following consent, block randomisation will be used to determine which participant

receives the intervention according to a computer-generated random assignment sequence prepared in advance by a statistician. The random assignment will be computer generated by a researcher not directly involved in the study and sixty opaque envelopes containing patient assignment will be prepared. Randomisation will be clustered into groups of random size of between 8 and 12 to ensure even distribution without bias. Only when a subject has fulfilled all study criteria will allocation take place. The patient's assignment will be performed by the data manager who will have no direct contact with the subject either before or after assignment. The clinicians involved in the assessment or treatment of patients will have no role in the assignment process. Using this process, we have successfully recruited patients for 3 completed randomised trials (19, 41, 48) as well as two continuing projects. Subjects randomised to the treatment arm will then be referred to CBS for assessment for LAGB. Subjects randomised to the control group will be referred back to orthopaedic pre-admission clinic at SVHM for routine work-up for TKA. We recognise that blinding of the surgical team to preoperative interventions may not be practically possible in light of the ethical need to obtain a full medical history. However, this should not preclude the provision of standardised surgical care. As CIB will be performing the LAGB procedures, she will not be involved in study data collection. Furthermore, outcome ascertainment will be blinded, and data will be analysed on an intention-to-treat basis.

4.6 Study Interventions:

Participants will be randomised to one of two groups:

(1) Intervention group: LAGB + TKA or
(2) Control group: TKA alone

4.6.1 Intervention group: LAGB+TKA

The treatment group will undergo LAGB followed by TKA 12 months later or after 20% loss of baseline body weight if this occurs earlier. We have demonstrated that a majority of weight loss occurs between 6 and 12 months post LAGB (19) (AI-O'Brien). We feel that delaying surgery for 6 to 12 months is justified if weight loss is equal to or in excess of 20% as this will be sufficient to reclassify a vast majority of patients to a BMI category that incurs a significantly lower complication rate (15). LAGB will be performed by surgeons affiliated with the Centre for Bariatric Surgery (CBS), with the surgery performed at The Avenue Hospital (Windsor, Victoria). The Centre has a proven track record in obesity surgery and in completing randomised trials involving LAGB (19, 41, 48). This will ensure that consenting patients are not disadvantaged by prolonged waiting times for LAGB in the public health system. Patients will attend monthly follow-up visits after LAGB, during which they will be clinically reviewed by the multidisciplinary team, weighed, receive dietary counselling and may have the volume of fluid in their lap band adjusted. Data are collected prospectively on a real-time web-based programme (Lap-Base™). Patients will be referred back to SVHM for their TKA at 12 months or after a 20% loss of baseline body weight if this occurs earlier. Weight at the time of surgery, change in BMI and body composition will be recorded at this time.

Surgery and care of patients undergoing primary TKA at SVHM is standardised through the use of clinical pathway protocols which have been validated in a randomised controlled trial (49). Only 2 prosthesis types will be used and there is no reported evidence that patient weight has a bearing on wear characteristics between these implants. Surgical technique is standardised.

4.6.2 Control Group: TKA alone

Patients in the control group will undergo TKA with routine follow-up and will not be offered specific management of their weight. This is in line with current practice. The literature has shown that dietary

interventions in obese patients with end-stage OA have resulted in only 4.9 to 8.7% weight loss (45, 46). Therefore we cannot justify delaying surgery in severely obese patients, when the percentage of weight loss will not sufficiently reduce BMI to a category that incurs a significant reduction in the complication rate (15).

4.7 Participant Follow Up

4.7.1 Intervention Group (LAGB + TKA) Follow Up

Patients will attend routine monthly follow-up visits after LAGB, at one of the satellite Centre for Bariatric Surgery (CBS) clinics located throughout metropolitan Melbourne, during which they will be clinically reviewed by the multidisciplinary team, weighed, receive dietary counselling, and may have the volume of fluid in their lap band adjusted. Patients will be referred back to SVHM to the Orthopaedic clinics for a review of their proposed TKA at 12 months or after a 20% loss of baseline body weight if this occurs earlier.

4.7.2 Control Group (TKA alone) Follow Up

Patients will undergo TKA with routine post-operative follow-up visits at SVHM at 6 weeks, 3 months and 12 months post-surgery and thereafter, follow-up occurs at 2, 5, 7 and 10 years.

4.8 Protecting Against Sources of Bias

We have carefully considered and identified possible sources of bias whereby we have addressed these in applying safe guards in order to mitigate such breeches.

(i) **Randomisation:** block randomisation will be used to determine which participant receives the intervention according to a computer-generated random assignment sequence prepared in advance by a statistician. The random assignment will be computer generated by a researcher not directly involved in the study and sixty opaque envelopes containing patient assignment will be prepared. Randomisation will be clustered into groups of random size of between 8 and 12 to ensure even distribution without bias.

(ii) **Pre TKA-Review:** We recognise that blinding of the surgical team to pre-operative interventions will not be possible in light of the ethical need to obtain a full medical history. However, orthopaedic surgeons performing TKA procedures will have not role in outcome ascertainment

(iii) **Blinding to the Adjudication of Outcomes:** Outcome ascertainment will be blinded, and data will be analysed on an intention to treat basis. The processes for identifying outcomes will be conducted by a Research Officer who will be blinded to the intervention allocation. The Research Officer will collate information on all possible primary outcomes of interest for review by an outcome's verification panel comprising at least 3 independent clinicians blinded to the patients' intervention allocation. Ascertainment of outcomes will be undertaken according to review against strict criteria (to be determined and endorsed by the panel).

5.0 Statistical Plan

5.1 Sample size determination

The choice of our sample size calculation was based on the following parameters:

(i) alpha value = 0.05, 2-sided;

(ii) power = 80%;

(iii) expected rates of the primary outcome (defined above) at 1 year post TKA of 8.8% for the LAGB+TKA group and 29.6% for the TKA alone group. The expected rates of the primary outcome were derived from existing data from the SVHM TKA database.

From 2006 to 2007, of the 118 patients (aged <70yrs) with BMIs of ≥35 kg/m2 who underwent primary TKA, 35 (29.6%) experienced the primary outcome. Hence this was the assumed rate for the TKA alone (control) group. LAGB is expected to reduce weight by 20% by 12 months (19). This amount of weight loss would have led to reclassification of the vast majority of the 118 patients to the BMI categories 25-30 and 30-34kg/m2. Hence the observed rate of the primary outcome among patients in these weight categories (12/137 = 8.8%) was assumed for the LAGB+TKA group. The sample size required in each of the 2 (equally-sized) groups is 55. To allow for drop-out of patients, we will recruit 120 patients in total. These numbers will also grant us more than 80% power to detect the expected differences in cumulative event-free survival between the 2 groups.

5.2 Statistical Analysis Plan

Statistical analysis will be by intention to treat. Categorical variables will be applied to chi-squared tests (or Fisher's Exact tests for small samples) while continuous variables will be applied to (parametric) t-tests and (non-parametric) Mann-Whitney tests for symmetrically and asymmetrically distributed data, respectively. Because time-to-outcome data will also be collected, survival analyses will also be undertaken. Cox proportional hazards regression analysis, or other types as appropriate to the data, will be used to derive hazard ratios associated with LAGB+TKA versus TKA alone.

5.2.1 Health Economic Analysis

We will apply health economic modelling under the direction of CIC to estimate the potential cost-effectiveness of LAGB+TKA. Decision analysis (52) will be used to compare the downstream consequences of LAGB+TKA versus TKA alone. The incorporation of Markov (53) and life-tabling (54) techniques will allow for the modelling of outcomes beyond one year. The main output of interest in health economic modelling is incremental cost-effectiveness ratios in terms of net costs per unit of health gain. Net costs will comprise the costs of LAGB+TKA minus costs saved from the reduction in downstream health services utilization. Health gains can be measured in a variety of ways. In our study, other than clinical outcomes, we will be also estimating years of life gained and quality-adjusted life years (QALYs) gained. Both are enabled by the collection of time-to-outcome data and the latter also by collection of QoL data. All health economic analyses will be undertaken in accordance with recommended approaches, such as 5% discounting of estimated future costs and health gains. To account for any uncertainty in the data inputs for health economic modelling, sensitivity and uncertainty analyses will be undertaken via Monte Carlo simulation (55).

5.2.2 Feasibility

This proposal represents an innovative clinical research program that brings together 3 major centres with recognised expertise in the management of two national health priorities: namely, OA and obesity. SVHM is a Victorian State Centre for joint replacement surgery that combines a leading academic Orthopaedic Department (chaired by CIA and backed by extensive surgeon experience, high volume throughput of elective joint replacement and a comprehensive arthroplasty database) with the infrastructure and expert academic support of the University of Melbourne (Departments of Surgery and Medicine). CID, an early career researcher, is an expert in conducting and co-ordinating randomised trials in joint replacement surgery. She has a deep understanding of obesity related outcomes in joint replacement after recently completing her PhD (NHMRC scholarship #502021) in the prognostic significance of obesity in joint arthroplasty. The Centre for

Obesity Research and Education (CORE), based at the Monash Medical School, Alfred Hospital, is headed by CIB. CIB and AI-Obrien are international leaders in obesity research and management and CORE is a world class collaboration of renowned international researchers and educators studying every facet of obesity. CIC is the Director of the Melbourne EpiCentre (Centre for Clinical Epidemiology, Biostatistics and Health Services Research) at the University of Melbourne and Melbourne Health. He is an epidemiologist with expertise in clinical trials and epidemiological and economic modelling. CIC's track record includes NHMRC and ARC funded projects and service as an expert advisor in a number of Commonwealth and State health committees. The combined expertise and resources of the research team creates a unique opportunity to examine the clinical and economic impact, as well as interdependence, of surgical treatments for obesity and osteoarthritis.

Under the supervision of CIA and CID, a high fidelity arthroplasty database containing epidemiologic and functional outcomes data has been constructed over the last 12 years. Currently, it houses over 7000 patients undergoing over 6000 procedures and represents a unique and valuable resource. The logistics of patient recruitment and follow-up for this study will be enhanced by the (deeply-entrenched) routine clinical and administrative practices existent at SVHM, which ensures a highly efficient elective surgical access scheme for joint replacement surgery. Over the last 5 years, 3000 patients have passed through this system. In a similar fashion, CIB and AI-O'Brien have been intimately engaged in the development of a lap-banding database dating back 15 years. They have developed an efficient system for the conduct of randomised trials of lap-banding surgery and have successfully published their results in high impact journals. The critical part of this project is the recruitment and follow-up of patients and both institutions have existing infrastructure and expertise to amalgamate and coordinate processes to facilitate the study.

The critical elements of a sound research plan with clear objectives, recognised expertise, established research infrastructure, track record and appropriate patient volume have been brought together in this proposal. The collaboration between two leading academic surgical groups makes the success of this project highly likely, and within the timelines proposed.

6.0 Data Collection and Management

6.1 Data Collection

All patients will have their initial weight and height recorded and in addition their demographics (sex, age and country of birth), co-morbidities, medical history and current medications taken during the consent process.

6.1.1 Patients randomised to the intervention group (LAGB + TKA), will attend monthly follow-up visits after LAGB, where they will have their weight and volume of fluid in their lap band adjusted and recorded. Data will be collected prospectively on a real-time web-based programme (Lap-Base™).

- **Anthropomorphic measurements** BMI and waist hip ratio measurements will be taken in the orthopaedic PAC, at 1-3 monthly intervals post LAGB, prior to and at 12 months post TKA.
- **Blood indices:** Blood for full blood examination, urea and electrolytes, renal function, measures of glucose intolerance, iron studies, vitamin B12 and D, folate, parathyroid hormone and calcium will be collected prior to and after LAGB and as part of routine monitoring pre and post this procedure.
- **Medications:** all medications and their doses will be tracked at monthly intervals post LAGB, prior to and at 12 months post TKA.

- **Function and QoL scores:** The Western Ontario and McMaster Universities Osteoarthritis Index (50) and SF-12 (51) questionnaires will be administered prior to LAGB and at 6, 12, 18 months and 2 years post LAGB. In addition prior to TKA surgery and annually post-operatively.
- **Radiology:** routine x-rays will be performed within 2 weeks prior to LAGB surgery, and 12 months post. This includes full-leg weight bearing radiographs enabling assessment of alignment. As is standard for patients undergoing TKA at SVHM routine x-rays will be performed within 2 weeks prior to surgery, post-operatively and at 12 months post-surgery. This includes full-leg weight bearing radiographs enabling assessment of prosthetic alignment.
- **TKA Surgery:** Patients will be referred back to SVHM for their TKA at 12 months or after a 20% loss of baseline body weight if this occurs earlier. Weight at the time of surgery, change in BMI and body composition will be recorded at this time. All post TKA data collection will be as stated below for routine TKA.

6.1.2 Patients randomised to the control group (TKA alone):

Will have and anaesthetic and surgery details on the day of surgery. All patients who have had their TKA the following data will be collected:

- **Anaesthetic and surgery details**: on the day of surgery
- **Bed utilization:** length of stay for the index surgery, in-patient rehabilitation and subsequent related admissions within the first 12 months
- **Anthropomorphic measurements** BMI and waist hip ratio measurements will be taken in the orthopaedic PAC at monthly intervals post LAGB, prior to and at 12 months post TKA.
- **Function and QoL scores:** The Knee Society score (50) and SF-12 (51) are both validated assessment tools for measuring health related QoL after TKA. These questionnaires will be administered prior to both LAGB and TKA procedures and annually after TKA.
- **Blood Indices:** routine blood indices full blood examination, urea and electrolytes, will be collected as clinically necessary deemed at pre-admission clinic.
- **Health services utilisation** Information regarding utilisation of health services, including medications and in- and outpatient services, will be captured by linkage of our database to administrative and clinical databases maintained by SVHM. The latter capture extensive information on *all* healthcare encounters occurring at SVHM's hospital network, including administered medications as well as fully-itemised costs of healthcare encounters.
- **Radiology:** As is standard for patients undergoing TKA at SVHM, routine x-rays will be performed within 2 weeks prior to surgery, post-operatively and at 12 months post-surgery. This includes full-leg weight bearing radiographs enabling assessment of prosthetic alignment.

6.2 Data Integrity

Study personnel will record data in a spreadsheet that is password protected to ensure and maintain patient confidentiality. Any missing, implausible or inconsistent data will be queried by Study Supervisors.

7.0 Ethical Considerations

This study is to be conducted in accordance to the National Health and Medical Research Council (NHMRC) guidelines and in addition to the international standards of Good Clinical Practice (GCP), applicable

government regulations, and institutional research policies and procedures. All study intervention and control phases fall within the spectrum of current standard practice, as do the standardised post-intervention phase follow-up visits.

7.1 Research Ethics Approval

The study proposal was submitted to the St Vincent's Ethics committee HREA for formal approval of the study conduct, and was granted final approval on the 19th January 2012. At each participating clinical site, the decision of the appropriate ethics committee concerning the conduct of the study will be made in writing to the local investigator. A copy of this decision will be provided to St Vincent's Hospital Human research ethics Committee-A using reference HREC-A 142/11 prior to the local commencement of this study.

7.2 Informed Consent

All patients eligible for this study will be provided with an Informed Consent Form describing this study and providing sufficient information for patients to make an informed decision about their participation in this study. The Informed Consent Form will comply with the NHMRC and SVHM HREC guidelines. The formal consent of a study participant, using the ethics committee-approved Informed Consent Form, must be obtained prior to the subject undergoing any study procedure.

7.3 Confidentiality

Information about study participants will be kept confidential and managed in accordance with the following NHMRC guidelines:

- All study-related information will be stored securely;
- All study participant information will be stored in locked file cabinets and accessible only to authorised study personnel;
- All study databases will be password-protected and accessible only to authorised study personnel

The communication and transmission of participant data will be de-identified in compliance with the SVHM HREC ethics committee. In the event that a participant revokes authorisation to collect or use personal health information, the participating clinical site retains the ability to use all information collected prior to the revocation of participant authorisation.

7.4 Protocol Amendments

Any amendments to the study protocol that may affect the conduct of the study or the potential safety of, or benefits to, participants (e.g., changes to the study objectives, study design, sample size, or study procedures) will require a formal amendment to the protocol. Any protocol amendments will need to be approved by the Principal Investigator, the SVHM HREC committee and local participating clinical sites ethics committees. If participating clinical sites request an amendment will also be required to submit amendment requests to their local ethics committees to obtain approval for the amendment, and to provide the SVHM HREC Committee with a copy of this approval. Administrative changes will also require formal approval for the amendment.

7.5 Safety and Adverse Events

Participating clinical sites are responsible for reporting all adverse event (AE) and serious adverse events (SAE) to SVHM research team and PI immediately and within in accordance with the NHMRC and SVHM HREC guidelines.

7.5.1 Clinical Site Notification

7.5.1.1 Notifying St Vincent's Hospital

Participating clinical sites are responsible for reporting AEs and SAEs to SVHM HREC committee. The original Adverse Event Forms will be kept on file in the relevant participant's file on site at SVHM.

7.5.1.2 Notifying the Appropriate Ethics Committee

Participating clinical sites are responsible for reporting SAEs and unanticipated problems resulting in risk to participants or others to their local ethics committee in accordance with local reporting requirements. Copies of each report and documentation of ethic committee notification and receipt will be kept in the participating clinical site's study file, and a copy sent to SVHM HREC committee.

8.0 Clinical Impact and Knowledge Dissemination

8.1 Potential Clinical Impact of the study

This project will exert a clinical impact if it shows that LAGB prior to TKA (i) increases the numbers of patients who have a sustained reduction in weight by at least 10%, which will lead to an overall improvement in cardiovascular and metabolic health; (ii) a reduces the numbers of patients experiencing complications following TKA, which will reduce the physical, personnel and financial resources required to treat these complications; (iii) increases the numbers of patients with measurable improvement in functional and QOL outcomes, which may result in reduce demands on carers and the community; and (iv) increases the numbers of patients who are deemed fit for TKA, which will reduce the symptomatic burden of pain, immobility and loss of independence. These data will lead to the development of evidence-based guidelines for the management of obese patients facing TKA, which will minimise the over- and under-treatment of obese patients afflicted with end-stage OA.

8.2 Realisation of the Clinical Impact

This project is due for completion 4 years after commencement. The feasibility of the project is high and it is anticipated that measurable outcomes will be realised on completion of the project. We expect that soon after completion of the project, important findings will be readily translated into practice through dissemination in the printed and electronic media, and at learned forums. We anticipate that the results of this project will inform the development of guidelines for the management of obese patients with end-stage OA.

8.3 Importance of Health Economic Modelling

Economic modelling is critical for the rational expenditure of health care resources. This is particularly relevant when dealing with a high volume, expensive procedure such as TKA. With 40,000 TKAs being undertaken annually at an estimated cost of $25000 each, the burden to funders approaches $1 billion annually. Understanding the variables that may influence this figure and whether LAGB, the most commonly performed (>90%) bariatric procedure in Australia (Medicare, 2007) may be deployed with clinical and financial benefit is important. This project will be the first to interrogate TKA and LAGB in this context.

8.4 Building Translational Research Capacity

This project will build translational research capacity by drawing on the expertise of the SVHM Department of Orthopaedics, CORE and The Melbourne EpiCentre to investigate a possible strategy for tackling a significant clinical and public health problem. Then, if appropriate, the collaboration could exploit their status as leading centres for TKA and LAGB to deploy this strategy. The potential for attracting doctoral and post-doctoral

researchers from a wide variety of biomedical sciences is also enhanced by the integration of the collaborating expert centres. These researchers will extend the research capacity and skills base of their disciplines with regard to obesity and OA management.

8.5 High Impact Publications

This is a first-of-its-kind project which is being conducted under rigorous conditions and with expert clinical and epidemiologic scrutiny. Several streams of data are anticipated including those related to TKA in obese patients, LAGB in TKA, LAGB in obese patients undergoing TKA, the outcomes (functional and QoL) of patients undergoing LAGB+TKA versus TKA alone, the economic analysis of TKA, and the economic analysis of LAGB+TKA and have significant potential for publication in high impact journals.

9.0 Study Timeline

Recruitment of 120 patients will occur during the first 2 years. TKA will be planned for 12 months following LAGB or after a 20% loss of baseline body weight if this occurs earlier. All TKAs will be performed by the 3rd year, with 12 month follow-up concluded by Year 4. The Gantt chart below details the time frame for each study phase. Longer term follow-up of study patients would provide valuable additional information about sustained changes and there is potential for follow-up to continue to 10 years post TKA.

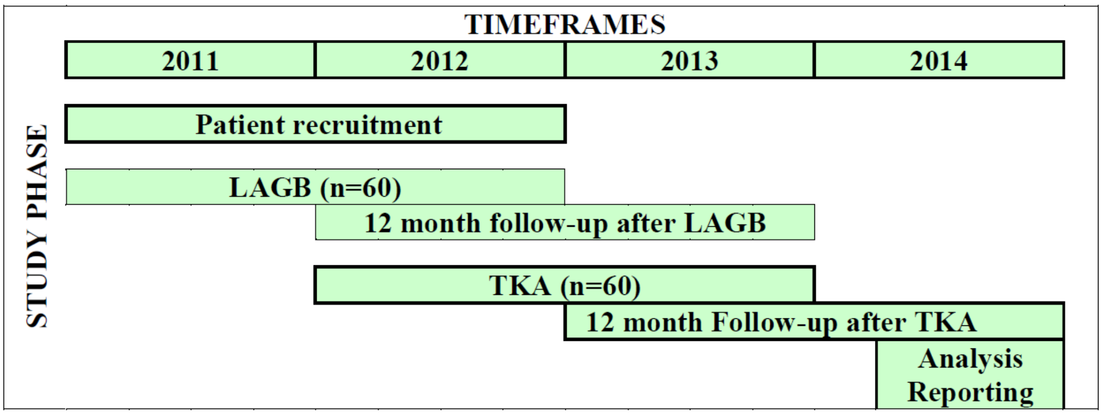

10.0 Reference List

1. World Health Organization. Obesity and Overweight. [Website] 2005 [cited 2007 31/12/2007]; Available from: www.infobase@who.int.

2. Forsight Report. Trends and Drivers of Obesity: A literature review for the Foresight project on obesity. 2007. p. 1-42.

3. Australian Orthopaedic Association. National Joint Replacement Registry Annual Report. Hip and knee replacement from September 1999 to December 2006. Adelaide:AOA. 2006 [30/12/2007]; Available from: www.dmac.adelaide.edu.au/aoanjrr/publications.jsp.

4. Sturmer T, Gunther KP, Brenner H. Obesity, overweight and patterns of osteoarthritis: the Ulm

Osteoarthritis Study. J Clin Epidemiol. 2000 Mar 1; 53(3):307-13.

5. Access-Economics. The economic costs of obesity2006.

6. Bourne R, Mukhi S, Zhu N, Keresteci M, Marin M. Role of Obesity on the Risk for Total Hip or Knee Arthroplasty. Clin Orthop Relat Res. 2007 Dec; 465:185-8.

7. Foran JR, Mont MA, Etienne G, Jones LC, Hungerford DS. The outcome of total knee arthroplasty in obese patients. J Bone Joint Surg Am. 2004 Aug; 86-A(8):1609-15.

8. Mantilla CB, Horlocker TT, Schroeder DR, Berry DJ, Brown DL. Risk factors for clinically relevant pulmonary embolism and deep venous thrombosis in patients undergoing primary hip or knee arthroplasty. Anesthesiology. 2003 Sep; 99(3):552-60; discussion 5A.

9. Miric A, Lim M, Kahn B, Rozenthal T, Bombick D, Sculco TP. Perioperative morbidity following total knee arthroplasty among obese patients. J Knee Surg. 2002 Spring; 15(2):77-83.

10. Namba RS, Paxton L, Fithian DC, Stone ML. Obesity and perioperative morbidity in total hip and total knee arthroplasty patients. J Arthroplasty. 2005 Oct; 20(7 Suppl 3):46-50.

11. Patel VP, Walsh M, Sehgal B, Preston C, DeWal H, Di Cesare PE. Factors associated with prolonged wound drainage after primary total hip and knee arthroplasty. J Bone Joint Surg Am. 2007 Jan; 89(1):33-8.

12. Vazquez-Vela Johnson G, Worland RL, Keenan J, Norambuena N. Patient demographics as a predictor of the ten-year survival rate in primary total knee replacement. J Bone Joint Surg Br. 2003 Jan; 85(1):52-6.

13. Winiarsky R, Barth P, Lotke P. Total knee arthroplasty in morbidly obese patients. J Bone Joint Surg Am. 1998 Dec; 80(12):1770-4.

14. Dowsey MM, Liew D, Stoney JD, Choong PF. The impact of obesity on weight change and outcomes at 12 months in patients undergoing total hip arthroplasty. Med J Aust. 2010 Jul 5; 193(1):17-21.

15. Dowsey MM, Liew D, Stoney JD, Choong PF. The impact of pre-operative obesity on weight change and outcome in total knee replacement: a prospective study of 529 consecutive patients. J Bone Joint Surg Br. 2010 Apr; 92(4):513-20.

16. Dowsey MM, Choong PF. Obese Diabetic Patients are at Substantial Risk for Deep Infection after Primary TKA. Clin Orthop Relat Res. 2008 Oct 8.

17. Buchwald H, Avidor Y, Braunwald E, Jensen MD, Pories W, Fahrbach K, et al. Bariatric surgery: a systematic review and meta-analysis. Jama. 2004 Oct 13; 292(14):1724-37.

18. O'Brien PE, Brown WA, Dixon JB. Obesity, weight loss and bariatric surgery. Med J Aust. 2005 Sep 19; 183(6):310-4.

19. O'Brien PE, Dixon JB, Laurie C, Skinner S, Proietto J, McNeil J, et al. Treatment of mild to moderate obesity with laparoscopic adjustable gastric banding or an intensive medical program: a randomized trial. Ann Intern Med. 2006 May 2; 144(9):625-33.

20. Kim SH. Morbid obesity and excessive hospital resource consumption for unilateral primary hip and knee

arthroplasty. J Arthroplasty. 2010 Dec; 25(8):1258-66.

21. Berend ME, Ritter MA, Meding JB, Faris PM, Keating EM, Redelman R, et al. Tibial component failure mechanisms in total knee arthroplasty. Clin Orthop Relat Res. 2004 Nov (428):26-34.

22. Ek ET, Dowsey MM, Tse LF, Riazi A, Love BR, Stoney JD, et al. Comparison of functional and radiological outcomes after computer-assisted versus conventional total knee arthroplasty: a matched-control retrospective study. J Orthop Surg (Hong Kong). 2008 Aug; 16(2):192-6.

23. Choong PF, Dowsey MM, Stoney JD. Does Accurate Anatomical Alignment Result in Better Function and Quality of Life? A Prospective Randomized Controlled Trial Comparing Conventional and Computer-Assisted Total Knee Arthroplasty. J Arthroplasty. 2008 May 17.

24. Browning RC, Kram R. Effects of obesity on the biomechanics of walking at different speeds. Med Sci Sports Exerc. 2007 Sep; 39(9):1632-41.

25. Lai PP, Leung AK, Li AN, Zhang M. Three-dimensional gait analysis of obese adults. Clin Biomech (Bristol, Avon). 2008; 23 Suppl 1:S2-6.

26. Segal NA, Yack HJ, Khole P. Weight, rather than obesity distribution, explains peak external knee adduction moment during level gait. Am J Phys Med Rehabil. 2009 Mar; 88(3):180-8; quiz 9-91, 246.

27. Astephen Wilson JL, Wilson DA, Dunbar MJ, Deluzio KJ. Preoperative gait patterns and BMI are associated with tibial component migration. Acta Orthop. 2010 Aug; 81(4):478-86.

28. Dowsey MM, Choong PF. Early outcomes and complications following joint arthroplasty in obese patients: a review of the published reports. ANZ J Surg. 2008 Jun; 78(6):439-44.

29. Nunez M, Nunez E, del Val JL, Ortega R, Segur JM, Hernandez MV, et al. Health-related quality of life in patients with osteoarthritis after total knee replacement: factors influencing outcomes at 36 months of follow-up. Osteoarthritis Cartilage. 2007 Sep; 15(9):1001-7.

30. National Health and Medical Research Council. Clinical Practice Guidelines for the management for overweight and obesity in adults.2003.

31. Colquitt J, Clegg A, Loveman E, Royle P, Sidhu MK. Surgery for morbid obesity. Cochrane Database Syst Rev. 2005(4):CD003641.

32. O'Brien PE, Dixon JB, Brown W, Schachter LM, Chapman L, Burn AJ, et al. The laparoscopic adjustable gastric band (Lap-Band): a prospective study of medium-term effects on weight, health and quality of life. Obes Surg. 2002 Oct; 12(5):652-60.

33. Christou NV, Sampalis JS, Liberman M, Look D, Auger S, McLean AP, et al. Surgery decreases long-term mortality, morbidity, and health care use in morbidly obese patients. Ann Surg. 2004 Sep; 240(3):416-23; discussion 23-4.

34. Dixon JB, Chapman L, O'Brien P. Marked improvement in asthma after Lap-Band surgery for morbid obesity. Obes Surg. 1999 Aug; 9(4):385-9.

35. Dixon JB, Dixon AF, O'Brien PE. Improvements in insulin sensitivity and beta-cell function (HOMA) with

weight loss in the severely obese. Homeostatic model assessment. Diabet Med. 2003 Feb; 20(2):127-34.

36. Dixon JB, Dixon ME, O'Brien PE. Depression in association with severe obesity: changes with weight loss. Arch Intern Med. 2003 Sep 22; 163(17):2058-65.

37. Dixon JB, O'Brien PE. Health outcomes of severely obese type 2 diabetic subjects 1 year after laparoscopic adjustable gastric banding. Diabetes Care. 2002 Feb; 25(2):358-63.

38. Dixon JB, O'Brien PE. Lipid profile in the severely obese: changes with weight loss after lap-band surgery. Obes Res. 2002 Sep; 10(9):903-10.

39. Dixon JB, O'Brien PE. Changes in comorbidities and improvements in quality of life after LAP-BAND placement. Am J Surg. 2002 Dec; 184(6B):51S-4S.

40. Dixon JB, Schachter LM, O'Brien PE. Polysomnography before and after weight loss in obese patients with severe sleep apnea. Int J Obes (Lond). 2005 Sep; 29(9):1048-54.

41. Dixon JB, O'Brien PE, Playfair J, Chapman L, Schachter LM, Skinner S, et al. Adjustable gastric banding and conventional therapy for type 2 diabetes: a randomized controlled trial. Jama. 2008 Jan 23; 299(3):316-23.

42. Lavernia C, Lee DJ, Hernandez VH. The increasing financial burden of knee revision surgery in the United States. Clin Orthop Relat Res. 2006 May; 446:221-6.

43. Parvizi J, Trousdale RT, Sarr MG. Total joint arthroplasty in patients surgically treated for morbid obesity. J Arthroplasty. 2000 Dec; 15(8):1003-8.

44. Christensen R, Astrup A, Bliddal H. Weight loss: the treatment of choice for knee osteoarthritis? A randomized trial. Osteoarthritis Cartilage. 2005 Jan; 13(1):20-7.

45. Messier SP, Loeser RF, Miller GD, Morgan TM, Rejeski WJ, Sevick MA, et al. Exercise and dietary weight loss in overweight and obese older adults with knee osteoarthritis: the Arthritis, Diet, and Activity Promotion Trial. Arthritis Rheum. 2004 May; 50(5):1501-10.

46. Miller GD, Nicklas BJ, Davis C, Loeser RF, Lenchik L, Messier SP. Intensive weight loss program improves physical function in older obese adults with knee osteoarthritis. Obesity (Silver Spring). 2006 Jul; 14(7):1219-30.

47. Moher D, Schulz KF, Altman D. The CONSORT statement: revised recommendations for improving the quality of reports of parallel-group randomized trials. JAMA. 2001 Apr 18; 285(15):1987-91.

48. O'Brien PE, Sawyer SM, Laurie C, Brown WA, Skinner S, Veit F, et al. Laparoscopic adjustable gastric banding in severely obese adolescents: a randomized trial. JAMA. 2010 Feb 10; 303(6):519-26.

49. Dowsey MM, Kilgour ML, Santamaria NM, Choong PF. Clinical pathways in hip and knee arthroplasty: a prospective randomised controlled study. Med J Aust. 1999 Jan 18; 170(2):59-62.

50. Bellamy N, Buchanan WW, Goldsmith CH, Campbell J, Stitt LW. Validation study of WOMAC: a health status instrument for measuring clinically important patient relevant outcomes to antirheumatic drug therapy in patients with osteoarthritis of the hip or knee. J Rheumatol. 1988 Dec; 15(12):1833-40.

51. Dunbar MJ, Robertsson O, Ryd L, Lidgren L. Appropriate questionnaires for knee arthroplasty. Results of a survey of 3600 patients from The Swedish Knee Arthroplasty Registry. J Bone Joint Surg Br. 2001 Apr; 83(3):339-44.

52. Lilford RJ, Pauker SG, Braunholtz DA, Chard J. Decision analysis and the implementation of research findings. BMJ. 1998 Aug 8; 317(7155):405-9.

53. Briggs A, Sculpher M. An introduction to Markov modelling for economic evaluation. Pharmacoeconomics. 1998 Apr; 13(4):397-409.

54. Wesley D. Life table analysis. J Insur Med. 1998; 30(4):247-54.

55. Briggs AH. Handling uncertainty in cost-effectiveness models. Pharmacoeconomics. 2000 May; 17(5):479-500.
