# Supplementary material for: Effect of Bariatric Surgery on Risk of Complications After Total Knee Arthroplasty: A Randomized Clinical Trial
Source: JAMA Netw Open. 2022 Apr 14;5(4):e226722. doi: 10.1001/jamanetworkopen.2022.6722 (PMC9011119; doi:10.1001/jamanetworkopen.2022.6722)
Supplement: Supplement 2. — eTable. Reason for Study Exclusion eFigure. Study Timelines [file jamanetwopen-e226722-s002.pdf]

Supplemental Online Content

Dowsey MM, Brown WA, Cochrane A, Burton PR, Liew D, Choong PF. Effect of bariatric surgery on risk of complications after total knee arthroplasty: a randomized clinical trial. *JAMA Netw Open*. 2022;5(4):e226722. doi:10.1001/jamanetworkopen.2022.6722

eTable. Reason for Study Exclusion

eFigure. Study Timelines

This supplemental material has been provided by the authors to give readers additional information about their work.

© 2022 Dowsey MM et al. *JAMA Network Open.*

eTable. Reason for Study Exclusion

Excluded	Number (n=277)
**Not meeting inclusion criteria**	**152**
• Non-English speaking	• 15
• Previous/current cancer diagnosis	• 14
• Mental health impairment	• 19
• Previous abdominal surgery	• 13
• Pervious bariatric surgery	• 30
• Medical condition	• 32
• Rural (limited access to services)	• 8
• Other	• 21
**Declined participation**	**125**
• Negative impression of LAGB	• 8
• Preference for medical management	• 32
• Travel burdensome	• 23
• Did not want to delay TKA	• 24
• Scheduled date for TKA	• 3
• Belief that weight loss was unnecessary	• 3
• Family declined participation	• 3
• GP declined participation	• 3
• Too stressful	• 7
• Virtual LAGB	• 2

© 2022 Dowsey MM et al. *JAMA Network Open.*

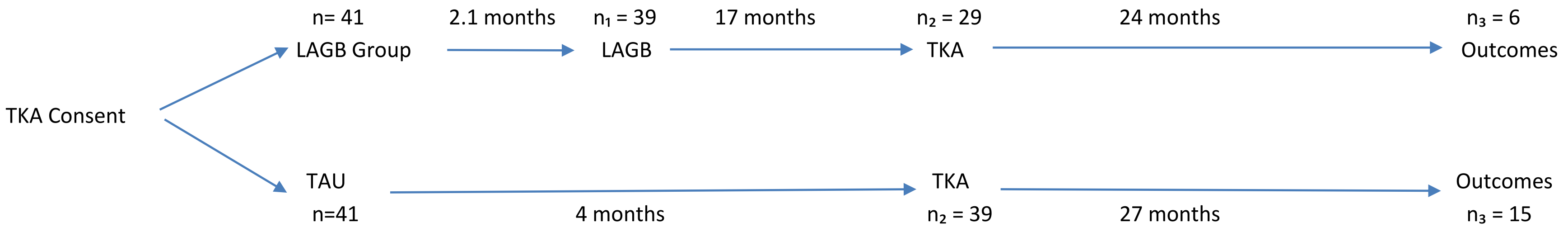

eFigure. Study Timelines

© 2022 Dowsey MM et al. *JAMA Network Open.*
