# Supplementary material for: Effect of Bariatric Surgery on Risk of Complications After Total Knee Arthroplasty: A Randomized Clinical Trial
Source: JAMA Netw Open. 2022 Apr 14;5(4):e226722. doi: 10.1001/jamanetworkopen.2022.6722 (PMC9011119; doi:10.1001/jamanetworkopen.2022.6722)
Supplement: Supplement 3. — Data Sharing Statement [file jamanetwopen-e226722-s003.pdf]

Data Sharing Statement

Dowsey. Effect of Bariatric Surgery on Risk of Complications After Total Knee Arthroplasty. *JAMA Netw Open*. Published April 14, 2022. doi:10.1001/jamanetworkopen.2022.6722

Data
Data available: No

Additional Information
Explanation for why data not available: Consent for data sharing was not obtained and ethics approval would be required from the study institutions for future use of trial data.
